# DISCUSSING SPIRITUAL HEALTH IN PRIMARY CARE IN ENGLAND

**DOI:** 10.1093/geroni/igz038.3237

**Published:** 2019-11-08

**Authors:** Ishbel Orla Whitehead

**Affiliations:** Newcastle University, Newcastle, United Kingdom

## Abstract

The organisation that regulates doctors and family physicians’ professional body in the UK both require doctors to consider patients’ spiritual health, especially towards the end of life. Discussion of spiritual health can encapsulate positive aspects of patients’ lives, and may be valuable for older people as physical and mental health decline. Tools are available for doctors to structure discussion of spiritual health in consultations but anecdotal reports suggest that this seldom happens. This study aimed to understand the barriers to GPs discussing spiritual health and their knowledge and views of current tools, particularly the HOPE tool by Anandarajah and Hight. Narrative literature review using systematic methods and mixed methods investigation into current practice, An online survey was conducted with 177 family physicians in England, investigating how doctors define spiritual health, their comfort with the topic, and their knowledge and acceptability of the HOPE tool, using patient vignettes. Definitions of spiritual health were heterogeneous, within three themes: self-actualisation and meaning; transcendence and relationships beyond self; and expressions of spirituality. Doctors felt more comfortable discussing spiritual health after a patient-led cue. Introduction of the HOPE tool increased doctors’ comfort with the topic. Discordance between doctor and patient beliefs and cultural backgrounds influenced views and practice. Concerns about regulator disapproval was a major barrier to discussions. Spiritual health does not appear to be a routine part of family practice in the UK. Tailored education, containing a structured tool such as HOPE, with regulatory approval, may help overcome barriers to discussion of spiritual health.

